# Assessment of the perceived safety culture in the petrochemical industry in Japan: A cross-sectional study

**DOI:** 10.1371/journal.pone.0226416

**Published:** 2019-12-12

**Authors:** Erman Çakıt, Andrzej Jan Olak, Atsuo Murata, Waldemar Karwowski, Omar Alrehaili, Tadeusz Marek

**Affiliations:** 1 Department of Industrial Engineering, Gazi University, Ankara, Turkey; 2 The Bronisław Markiewicz State Higher School of Technology and Economics, Jarosław, Poland; 3 Department of Intelligent Mechanical Systems, Graduate School of Natural Science and Technology, Okayama University, Okayama, Japan; 4 Department of Industrial Engineering and Management Systems, University of Central Florida, Orlando, FL, United States of America; 5 Department of Industrial Engineering, Taibah University, Madinah, Saudi Arabia; 6 Institute of Applied Psychology, Jagiellonian University, Krakow, Poland; Fukushima Medical University School of Medicine, JAPAN

## Abstract

This study assessed the perceived safety culture among five petrochemical production companies in Japan. Current effects of the perceived safety culture on employee safety motivation and performance were also examined. A total of 883 workers from the five petrochemical companies, which were located in the Chugoku region of Japan, provided valid responses to the survey distributed by email. Structural equation modeling was used to evaluate the personnel safety culture in these industries. The endogenous variables considered in this study included petrochemical safety culture, personnel error behavior and personnel attitudes toward violation behaviors. Petrochemical personnel safety motivation was a mediating variable. This study’s findings highlight the importance of the perceived safety culture as a significant component of the organizational culture that influences employee behaviors and safety attitudes. This study further verifies the significant impact of the perceived safety culture in this industry sector on improving petrochemical personnel safety motivation and performance. Future research should explore the differences between the subcultures that have formed under larger safety cultures within similar high-risk industries, such as construction, aviation, manufacturing and mining.

## Introduction

In a recent study on managing the risks of organizational accidents, Reason (2016) argued that the same general safety principles and management techniques can be applied in various domains, such as banks, insurance companies, nuclear power plants, oil exploration, manufacturing companies, chemical process installations and other domains of industry, transportation and healthcare [1]. The International Nuclear Safety Advisory Group of the International Automatic Energy Agency defines a safety culture as “that assembly of characteristics and attitudes in organizations and individuals, which establishes that, as an overriding priority, nuclear plant safety issues receive the attention warranted by their significance” [2,3]. Consequently, a safety culture does not focus solely on safety attitudes; rather, it is a positive indicator of safety management performance. Furthermore, a safety culture that is rated as excellent assigns the highest priority to safety [4]. Most subsequent definitions have focused on the human beliefs, perspectives and behaviors in an organization [5]. The most widely cited definition of an organizational safety culture was developed by the Health and Safety Commission (1993) and published in the Advisory Committee on the Safety of Nuclear Installations report [6]. This report describes a safety culture as “the product of individual and group values, attitudes, perceptions, competencies and patterns of behavior that determine the commitment to, and the style and proficiency of, an organization’s health and safety management.” Prior research also confirms that a safety culture fosters risk management and mitigation strategies based on an increased commitment to and knowledge of safety within an organization. The observed outcome has been an increased readiness for potentially dangerous situations [7,8,9].

An inverse relationship has also been identified between safety culture and the occurrences of accidents and injuries in highly hazardous fields, such as the petrochemical industry. Consequently, the development and maintenance of a positive safety culture can be an effective tool for improving overall safety within an organization [10]. There is currently an urgent need for the management support of safety issues to foster a positive safety culture. This approach ensures accountability and develops workers who are fully informed regarding safety procedures and the importance of adhering to such procedures.

In the present study, the perceived safety culture in the petrochemical industry was captured through five main factors, including 1) management commitment toward safety, 2) employees’ personal attitude toward safety, 3) coworkers’ support of safety, 4) workplace pressure and 5) safety management system. Three different types of surveys can be used to measure the factors of safety culture. Three of the five safety culture components mentioned were measured using the survey in [11], which includes management’s commitment toward safety, coworkers’ support of safety and workplace pressure. Employees’ personal attitude toward safety was measured using a survey from [12]. Lastly, safety management system was measured using the survey in [13], in which the questions were created specifically for assessing safety culture.

The fourth survey used in this study was adopted from [14] and was designed to measure workers’ safety motivation to follow safety rules. The survey was used in previous research to measure the level of motivation that employees feel regarding and importance that employees place on following safety procedures and policies. Workers’ attitude toward violations was measured using survey questions [15]. The adopted survey has nine questions regarding workers’ attitude toward their own safety violation behavior. However, only five questions were selected due to the similarities among questions and to avoid repeated or unclear questions. Lastly, the fifth survey adopted in this study measures workers error behaviors [11]. We selected four questions to assess error behaviors with regard to skills, decision-making and error perceptions. Thus, this study is focused on answering the following questions:

Q1: What is the impact of the perceived safety culture on the safety motivation of personnel in the petrochemical industry in Japan?
Q2: What is the effect of perceived safety culture on personnel error behaviors in the petrochemical industry in Japan?
Q3: What is the effect of the existing perceived safety culture on personnel attitudes toward violations in the petrochemical industry in Japan?
Q4: Does personnel safety motivation in the Japanese petrochemical industry mediate the relationship between perceived safety culture and personnel error behaviors?
Q5: Does personnel safety motivation in the Japanese petrochemical industry mediate the relationship between perceived safety culture and personnel attitudes toward behavior related to safety violations?

## Theoretical background

### Safety culture applications

Safety culture has recently been the focus of extensive research in Japan’s healthcare industry. For example, Itoh and Andersen (2008) reported the results of a questionnaire-based survey on safety culture that included more than 20,000 staff responses from multiple Japanese hospitals [16]. The study identified basic characteristics of safety culture in Japanese healthcare, including differences in professional, regional and organizational cultures. Wu et al. (2013) investigated safety culture in the nursing profession based on a cross-national hospital survey on patient safety culture in Japan, the United States and Chinese Taiwan [17].

Safety culture has also received worldwide consideration in the nuclear industry [18]. For example, Takano et al. (2001) investigated the safety culture in nuclear power operations with a focus on the interrelationships between organizational factors and major safety indicators [19]. Recently, safety culture received renewed attention in Japan after the Fukushima Daiichi Nuclear Power Plant disaster of 2011 [20,21,22,23,24,25,26,27].

With respect to the petrochemical industry, the current English language literature on safety culture in Japan’s petrochemical industry is limited. Hsu et al. (2008) conducted a cross-cultural study of organizational factors that are related to safety in Japanese and Taiwanese oil refinery plants [28]. Structural equation modeling (SEM) was used to investigate the relationships between organizational factors and the safety performance of workers. The results indicated that organizational safety factors differ between Japan and Taiwan. Taiwanese plants are characterized by higher levels of management commitment to safety, a greater emphasis on safety activities and an increased devotion to supervision. Japanese plants exhibit higher levels of employee empowerment and stronger attitudes toward continuous improvement, a greater emphasis on a systematic safety management approach and efficient reporting systems and teamwork. The observed differences are primarily due to the respective levels of economic development between these two countries.

### Safety culture in the petrochemical industry

The petrochemical industry consists of three sectors: upstream, midstream and downstream. All three sectors expose employees to high-risk working environments. The upstream sector includes the manufacturing of basic raw materials, the midstream sector handles the manufacturing of intermediates, and the downstream sector manages the process and manufacturing of various byproducts [29]. A worker in this field may be exposed to a wide range of occupational hazards, e.g., fires, explosions, toxins, illnesses and other job-related risks in the manufacturing, transport and storage of petrochemical materials. Consequently, there is a need to understand how safety culture influences petrochemical personnel safety behaviors and performance in attempts to reduce hazards and ensure safe operations. Assessing safety culture in the petrochemical industry is a necessary step toward improving the overall safety performance in a highly hazardous field while enhancing future organizational success. The ultimate goal is to achieve effective management of safety performance to support workers in this complex, hazardous field.

Safety climate analysis through surveys may identify relationships between important safety dimensions within an organization and how they can contribute to overall safety culture [4]. Hosny et al. (2017) conducted a comparative analysis of workers’ perceptions of safety climate dimensions among three Egyptian petrochemical companies [30]. Worker involvement was observed as the primary factor in establishing a proper safety climate. The study also revealed significant differences in the workers’ perceptions of safety management applications among the three companies, especially in the worker involvement domain. Kao et al. (2008) identified eight safety culture factors in a particular petrochemical organization: safety commitment and support, safety attitude and behavior, safety communication and involvement, safety training and competence, safety supervision and auditing, safety management system and organization, accident investigation and emergency planning and, finally, reward and benefits [31]. The study concluded that personal backgrounds (e.g., job position, work experience and age) significantly affect perceptions in many safety culture dimensions.

Pordanjani and Ebrahimi (2015) investigated the relationships of safety motivation and work pressure with occupational accident rates among workers at the Khorasan petrochemical company in Iran [32]. This study demonstrated that safety motivation and work pressure are important predictors of the occupational accident rate. Salleh (2000) investigated safety behavior in the Malaysian petrochemical industry and concluded that safety motivation and employee conscientiousness and competency are positively and significantly related to safety behavior [33]. Safety commitment also partially mediated the relationship between safety motivation and employee conscientiousness and competency with safety behavior.

The literature review demonstrates an unresolved debate as to whether an organization is a culture by itself or has a specific culture related to it. There is no universally accepted model of safety culture [34]. One approach is the safety culture maturity model. Filho et al. (2010) developed a framework for examining and determining safety culture maturity levels within the Brazilian petrochemical industry [35]. Similarly, Boughaba et al. (2014) identified training, incentives, communication, manager commitment and employee involvement as having the greatest impact on a company’s safety culture maturity and safety performance [2]. The study further demonstrated how its safety culture influences its safety performance, which was confirmed by comparing two petrochemical plants within one company. Additionally, Wu et al. (2009) explored the causes and consequences of safety culture in the petrochemical sector [36]. Three scales have been developed: the safety leadership scale, the safety climate scale and the safety performance scale. Similarly, Shirali et al. (2016) applied an exploratory factor analysis to identify the current weaknesses and challenges that the petrochemical industry faces in creating a resilient safety culture [37].

### Factors affecting safety culture

Safety culture represents an organization’s safety practices and management, and safety culture may positively or negatively impact worker behavior. The measurement of an organization’s safety climate and culture can predict and serve as a feedforward type of control, as opposed to merely a feedback, lagging or inactive measure [38]. “Safety culture” became a popular term as a result of its ability to capture all important aspects of safety practices, including safety management systems, safety perception and safety behaviors. Management commitment combined with safety polices directly influence safety climate and culture [39]. In particular, management commitment has the strongest influence over safety culture as an outcome [40,41,4,42,43]. Therefore, safety culture is the primary factor of influence on employee attitudes and behaviors toward an organization’s ongoing safety performance and is effectively correlated to organizational culture. Consequently, safety culture has drawn the attention of a wide range of industries [34].

The factors that affect safety culture are classified into two main categories: organizational and social. Organizational factors exist within the organization itself and relate to the project situation, management style in the safety administration, safety attitudes, communication, group norms, ethnic diversity of workers, safety enforcement and control. Social factors reside outside the organization. These include governmental rules, society’s safety awareness and the impact of local culture on safety culture. Over the past two decades, a number of safety culture reciprocal models have been developed. These models include a multifactor analysis, reciprocal safety culture, nation-specific safety culture and theoretical safety culture. Wu et al. (2011) investigated the relationship among safety leadership, safety climate and safety performance using SEM [44]. Their results suggested that the safety climate mediates the relationship between safety leadership and performance.

## Objectives

Although safety culture has been studied extensively in certain areas, only limited information and assessments have been adopted with regard to following safety behaviors in the Japanese petrochemical industry. The assessment of an industry’s perceived safety culture is a crucial step toward identifying opportunities for safety performance and ultimately enhancing organizational success within the industry. An understanding of how perceived safety culture affects personnel safety performance and behaviors is required to reduce hazards and ensure safe operations. Therefore, the main objective of this study was to assess the perceived safety culture among five petrochemical companies in Japan and to identify safety culture development opportunities and potential safety performance improvements. The effects of the current safety culture on personnel safety motivation and performance were also assessed.

## Methods and procedures

### Study design

In this cross-sectional study, five primarily petrochemical-producing companies located in the Chugoku region of Japan participated in a survey on perceived safety culture. The safety manager at each company distributed the questionnaire to plant workers. A cover letter with the questionnaire contained both an invitation to participate and the informed consent form required for issuing the survey. The survey questionnaire and the experimental protocol for this study were approved by the Institutional Review Board (# FWA00000351, IRB00001138) at the University of Central Florida, Orlando, Florida, USA. Additionally, according to the rules of the ethical committee of the Okayama University in Japan, this questionnaire study was exempted from institutional review. However, all of the participating companies in Japan provided the required approval for conducting the study at their sites, and the participation of employees in Japan was voluntary and was conducted using the survey questionnaire and experimental protocol approved by the University of Central Florida. To our knowledge, there are no regulations associated with foreign researchers conducting questionnaire surveys in Japan. The questionnaire developed was validated on a small group of graduate students in the USA. The questionnaire was then validated on a large group of workers at construction sites in Saudi Arabia [45]. The original questionnaire was published in a dissertation by Alrehaili (2016) [45] and was copyrighted there. This questionnaire was translated into Japanese and then was first assessed by a small group of Japanese university researchers who are experts on the safety culture in the petrochemical industry. Next, a preliminary testing of the questionnaire using a small group of Japanese university students who had a broad knowledge of human factors and safety engineering was conducted. This validation step confirmed that all of the statements in the translated version of the survey were addressed by the students without any difficulties.

One of the authors (the Japanese researcher) met with the managers who were responsible for safety in the participating companies and explained the aim of the intended study. He also explained the questionnaire survey and data collection methodology. The management of each company agreed to facilitate in executing the survey. The managers asked the workers for voluntary participation in the study and allowed all volunteers to participate during their normal working hours. The survey was distributed electronically (e-mail) to all workers who agreed to participate in the survey. The worker responses were collected anonymously and, while maintaining anonymity, were coded into an Excel file that was sent by each company to the Japanese researcher.

### Study variables

The questionnaire was divided into two parts. The first part requested participant demographic information, e.g., age, gender, education, position and work experience. The second part included questions with responses that were measured on a five-point Likert scale that ranged from “1 = strongly disagree” to “5 = strongly agree.” To handle missing and unanswered data, surveys from participants who did not fully complete all survey statements were excluded by the companies and not included in the final data set. Given the above questionnaire information, the initial set of variables used in the model development is defined in Table 1.

**Table 1:** Model constructs and their corresponding item measures.

Construct and item measure description	
**Construct 1: Management commitment (MC)**	
**MC1**	The company’s management provides efficient work safety training for workers
**MC2**	If I report a mistake to my supervisor, management supports me
**MC3**	Management encourages workers to report every incident about safety to a supervisor
**MC4**	Management strongly supports safety for workers
**MC5**	Managers support work safety even if it causes a delay in work
**MC6**	My managers sometimes ignore work safety violations
**MC7**	My managers frequently speak unofficially with workers about safety
**MC8**	My management allows workers to work by being sensitive to safety rules
**MC9**	My supervisor gives importance to my opinion for improving work safety
**Construct 2: Employees personnel attitude (EPA)**	
**EPA1**	Work safety rules provide a safer work environment
**EPA2**	I make sure to use necessary safety equipment
**EPA3**	I alert my colleagues who act contrary to work safety rules
**EPA4**	If my colleagues do not take any notice, I notify my manager about unsafe work
**EPA5**	I try to follow work safety rules, even if they decrease my performance
**EPA6**	It is more likely to have an accident in a workplace where there are no work safety rules
**EPA7**	Work safety rules are important and necessary to prevent accidents at my work
**Construct 3: Coworkers safety support (CSS)**	
**CSS1**	Most workers notify personnel who are taking risks
**CSS2**	Most workers support workplace safety policies
**CSS3**	My colleagues usually suggest that I ignore work safety rules
**CSS4**	My colleagues point out each other’s deficiencies in work safety
**CSS5**	My colleagues want to help each other with work safety
**CSS6**	My colleagues attach importance to the assessment for incidents that can cause accidents
**Construct 4: Workplace pressure (WP)**	
**WPP1**	Completing work is more important than doing work in safe ways
**WPP2**	I sometimes compromise on safety to finish the work on time
**WPP3**	Sometimes, it is expected from me to do more work than to do it safely
**WPP4**	It is difficult to work when applying all work safety rules
**WPP5**	In my workplace, cutting corners and risky attitudes are common because of the heavy workload
**WPP6**	I am sometimes not sure if work can be done by following work safety rules
**WPP7**	I can easily get necessary safety equipment from my workplace
**Construct 5: Safety management system (SMS)**	
**SMS1**	Safety feedback and comments are always presented from and to management
**SMS2**	There is an understanding that workers will be thanked for their safety performance
**SMS3**	My company often offers safety incentives to site managers, site personnel and project engineers
**SMS4**	Safety rewards presented by my company are valuable
**SMS5**	Safety responsibility and accountability are clearly described
**SMS6**	Site managers and field personnel place importance on safety
**SMS7**	There are dedicated safety agents, and they usually observe and correct field personnel’s unsafe acts
**SMS8**	Field personnel are aware that unsafe performance will be punished and not tolerated
**SMS9**	Unsafe performance is consistently punished with reasonable levels that fit the violation
**SMS10**	Safety is always reinforced, even if a violation occurred without accident
**SMS11**	Management places importance on safety, and it is a strategic concern for top management
**SMS12**	Everyone is responsible for safety, not just safety staff
**SMS13**	My company policies and actions demonstrate a sincere commitment to safety
**SMS14**	Hazard analysis, prevention and control are very important and often performed at the petrochemical site
**SMS15**	Unsafe behavior identification with necessary corrections is often performed
**Construct 6: Violation behavior (VB)**	
**VB1**	I feel it is essentially important to maintain safety at all times
**VB2**	I believe safety in the workplace is a key issue
**VB3**	I feel that it is compulsory to expend effort to decrease accidents and incidents at my workplace
**VB4**	I feel it is important to encourage others to use safety practices
**VB5**	I feel it is important to promote safety programs
**Construct 7: Personnel safety motivation (PSM)**	
**PSM1**	I am capable of following all safety regulations and procedures
**PSM2**	It is clear to me how to follow work safety rules and procedures
**PSM3**	I have made safety errors due to not knowing how to work safely
**PSM4**	I have rarely made errors that caused risks in working
**Construct 8: Personnel error behavior (PEB)**	
**PEB1**	I carefully follow work safety rules and procedures when assigned a petrochemical task
**PEB2**	I can perform a task with which I am familiar without looking at written procedures and manuscripts
**PEB3**	I intentionally bend formal procedures to finish work on time
**PEB4**	I have ignored some parts of procedures and do not record these to make work easier in abnormal circumstances
**PEB5**	I am conscious of my responsibility about work safety

### Study hypotheses

This study’s hypotheses are based on several safety-relevant variables, including (1) management’s commitment to safety, (2) employee attitudes toward safety, (3) coworker’s support of safety, (4) work pressure and (5) plant safety management systems. The objective is to assess the impact of the perceived safety culture on personnel safety motivation and safety performance in the petrochemical environment. This approach is consistent with a heavy literary emphasis on the significant effects that organizational culture has on employee motivation [42,46].

**Fig 1:**
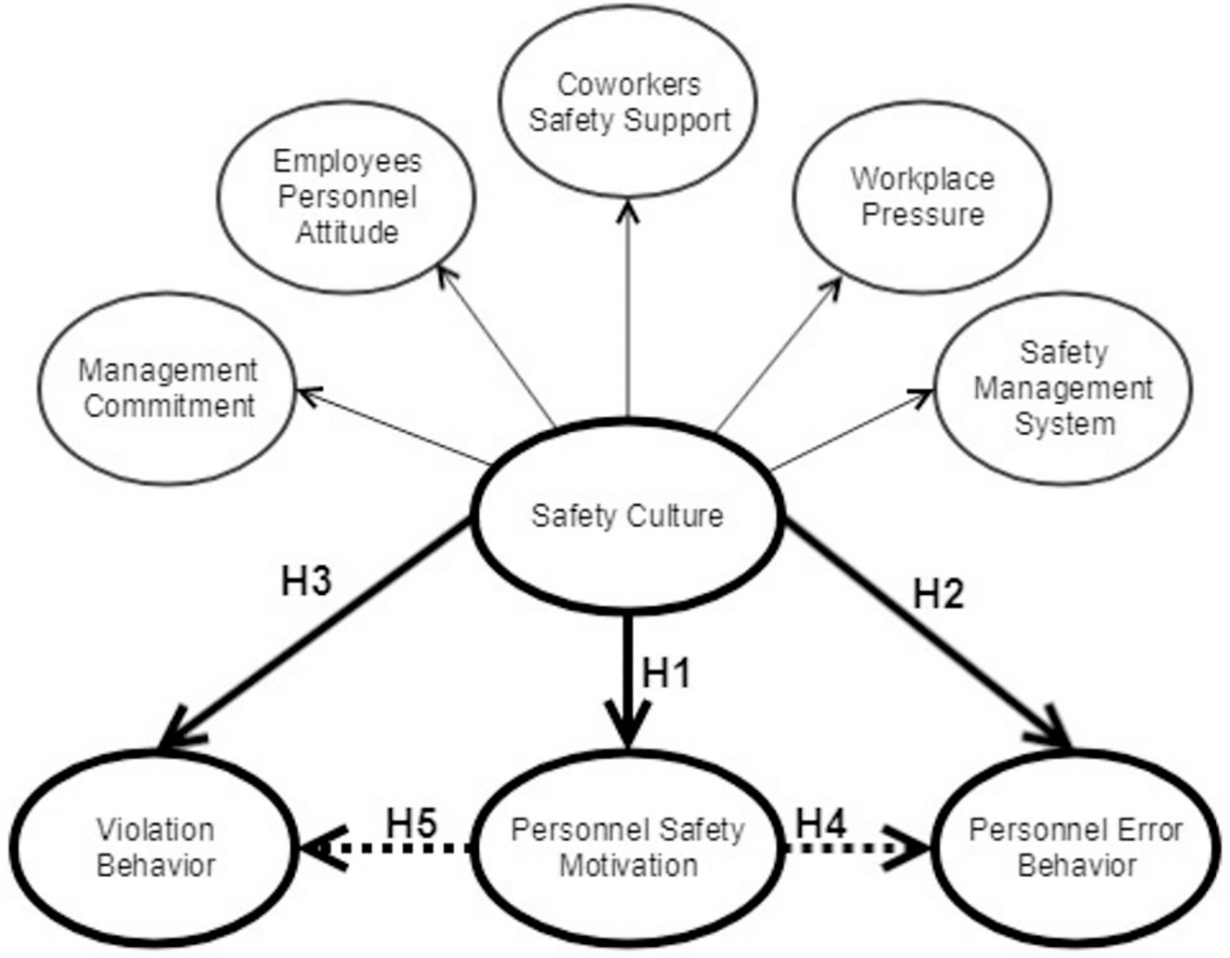
The hypothesized conceptual model.

The postulated hypotheses and their interrelationships are illustrated by the proposed model shown in Fig 1. The model attempts to evaluate the relationships between (1) perceived safety culture and (2) personnel safety motivation, (3) personnel error behavior and (4) attitudes toward violations. The model further examines the petrochemical industry’s safety culture among engineers, supervisors, safety officers and project managers. The objective in formulating the model was to offer a mechanism for forecasting safety performance as well as to evaluate the role of petrochemical safety personnel in mediating the perceived safety culture and safety performance.

The first hypothesis (H1) suggests a significant impact of the perceived safety culture in the petrochemical industry on worker safety motivation.

*H1*: *Perceived safety culture affects personnel safety motivation in petrochemical industries*.

Organizational factors, such as management’s commitment to safety [47,15] and safety culture perception [15], significantly affect employee error. Consequently, the second (H2) and third (H3) hypotheses posit the significant effects of perceived safety culture on employee error behaviors and their attitudes toward similar behaviors in the petrochemical industry. These hypotheses address the role of personnel safety motivation in mediating the perceived safety culture and safety performance.

*H2*: *Perceived safety culture affects personnel error behaviors in petrochemical industries*.
*H3*: *Perceived safety culture affects personnel attitudes toward violations in petrochemical industries*.

In these industries, safety performance is assessed quantitatively using worker error behavior and respective attitudes. The motivation of personnel to comply with safety rules and requirements is an essential component of enhancing safety performance [34,48]. Safety culture typically plays a crucial role in influencing worker safety motivation. Therefore, the fourth hypothesis (H4) investigates the influences of personnel safety motivation as a mediator between perceived safety culture and worker error behaviors in the petrochemical industry. Both the fourth and fifth hypotheses examine the effects of employee safety motivation as a mediator between perceived safety culture and worker attitudes toward violations.

*H4*: *Personnel safety motivation mediates the relationship between perceived safety culture and employee error behaviors in petrochemical industries*.
*H5*: *Personnel safety motivation mediates the relationship between perceived safety culture and employee attitudes toward violations in petrochemical industries*.

### Survey questionnaire

All questionnaire statements were measured on a five-point response Likert scale of (1) strongly disagree, (2) disagree, (3) neither agree nor disagree, (4) agree and (5) strongly agree. The order of statements in the questionnaire was kept the same throughout the study.

### Participants

A total of 1,456 workers have been invited to participate in the study. Of these, 883 returned the complete and valid surveys, resulting in the response rate of 60.6%. Furthermore, 99% of the participants were male workers. The age distribution included 134 (15.2%) respondents under the age of 26, 148 (16.8%) between 26 and 30, 80 (9.1%) between 31 and 35, 66 (7.5%) between 36 and 40, 112 (12.7%) between 41 and 45, and 343 (38.8%) over 45. Regarding work experience, 167 (18.9%) of the respondents had worked less than 5 years, 216 (24.5%) between 6 and 10, 60 (6.8%) between 11 and 15, 77 (8.7%) between 16 and 20, and 363 (41.1%) more than 21 years (Table 2). With regard to education level, 648 (73.4%) of the respondents had graduated from high school, 168 (19%) were college graduates, and 33 (3.7%) had earned a master’s degree. The remaining thirty-four participants (3.9%) had not completed high school. Additionally, 649 (73.5%) of the participants were project managers, 80 (9.1%) were supervisors, 64 (7.2%) were engineers, 17 (1.9%) were safety engineers, and 73 (8.3%) reported associations with other professions. Demographic information was compiled using IBM SPSS Version 25 for Windows (SPSS Inc., Chicago, IL, USA), and other statistical analyses were performed using the SmartPLS (v.3.2.8) software [49,50]. Multicollinearity analysis, testing the reliability, validity, path coefficients, and SEM were used to analyze the relationships among model factors.

**Table 2:** Profile of respondents.

Demographic variable	All (N = 883)	
	Frequency	(%)
Gender		
1. Male	874	99
2. Female	9	1
Age		
1. Less than 26	134	15.2
2. 26–30	148	16.8
3. 31–35	80	9.1
4. 36–40	66	7.5
5. 41–45	112	12.7
6. Older than 45	343	38.8
Work experience		
1. Less than 5 years	167	18.9
2. 6–10 years	216	24.5
3. 11–15 years	60	6.8
4. 16–20 years	77	8.7
5. More than 21 years	363	41.1

## Model development and analysis

### Multicollinearity analysis

We estimated the means and standard deviations for all study variables. Correlation analysis was also conducted to assess the relationship between any two variables used in model construction (Table 3). All model variables had significant relationships at *p* ≤ 0.01. In either a reflective or a formative model, there is potential multicollinearity at the structural level. Multicollineality was verified by an indicator of variance inflation factors. We used SmartPLS (v.3.2.8) to calculate the variance inflation factor (VIF) for all of the exogenous variables in the data group. According to Hair et al. (2016) [50], all VIFs were less than 5.0 (< 5.0) and thus were deemed to be acceptable measures. In other words, a common rule of thumb is that problematic multicollinearity may exist when the VIF coefficient is greater than 5.0. In this study, none of the VIF coefficient values exceeded the threshold value of 5.0, thus confirming that multicollinearity was not present in the model data.

**Table 3:** Means, standard deviation and correlations.

Constructs	Mean	S.D.	MC	EPA	CSS	WP	SMS	VB	PSM	PEB
**MC**	3.85	0.53	-	-	-	-	-	-	-	-
**EPA**	3.88	0.49	0.56	-	-	-	-	-	-	-
**CSS**	3.70	0.48	0.68	0.58	-	-	-	-	-	-
**WP**	1.56	0.52	-0.54	-0.53	-0.49	-	-	-	-	-
**SMS**	4.12	0.53	0.64	0.64	0.72	-0.61	-	-	-	-
**VB**	4.48	0.43	0.45	0.45	0.39	-0.45	0.59	-	-	-
**PSM**	1.38	0.74	-0.31	-0.32	-0.33	0.49	-0.38	0.28	-	-
**PEB**	2.88	0.41	0.53	0.57	0.57	-0.62	0.67	0.58	-0.48	-

*Notes*: Correlations are significant at *p*≤0.01

Abbreviations

Management commitment (MC); employees personnel attitude (EPA); coworkers safety support (CSS); workplace pressure (WP); safety management system (SMS); violation behavior (VB); personnel safety motivation (PSM); personnel error behavior (PEB).

### Reliability and convergent validity

SmartPLS (version 3.2.8) was conducted for testing the reliability, validity and path coefficients of our proposed model. For reliability, we used the Cronbach alpha and composite reliability as proposed based on the Fornell and Larcker’s (1981) [51] and Cronbach (1951) [52] criteria. For validity, we used convergent validity and discriminant validity, which are part of construct validity. The convergent validity describes the degree to which scale items truly represent the latent construct [53]. To establish convergent validity, we used Fornell and Larcker’s (1981) [51] criteria of the average variance extracted (AVE).

**Fig 2:**
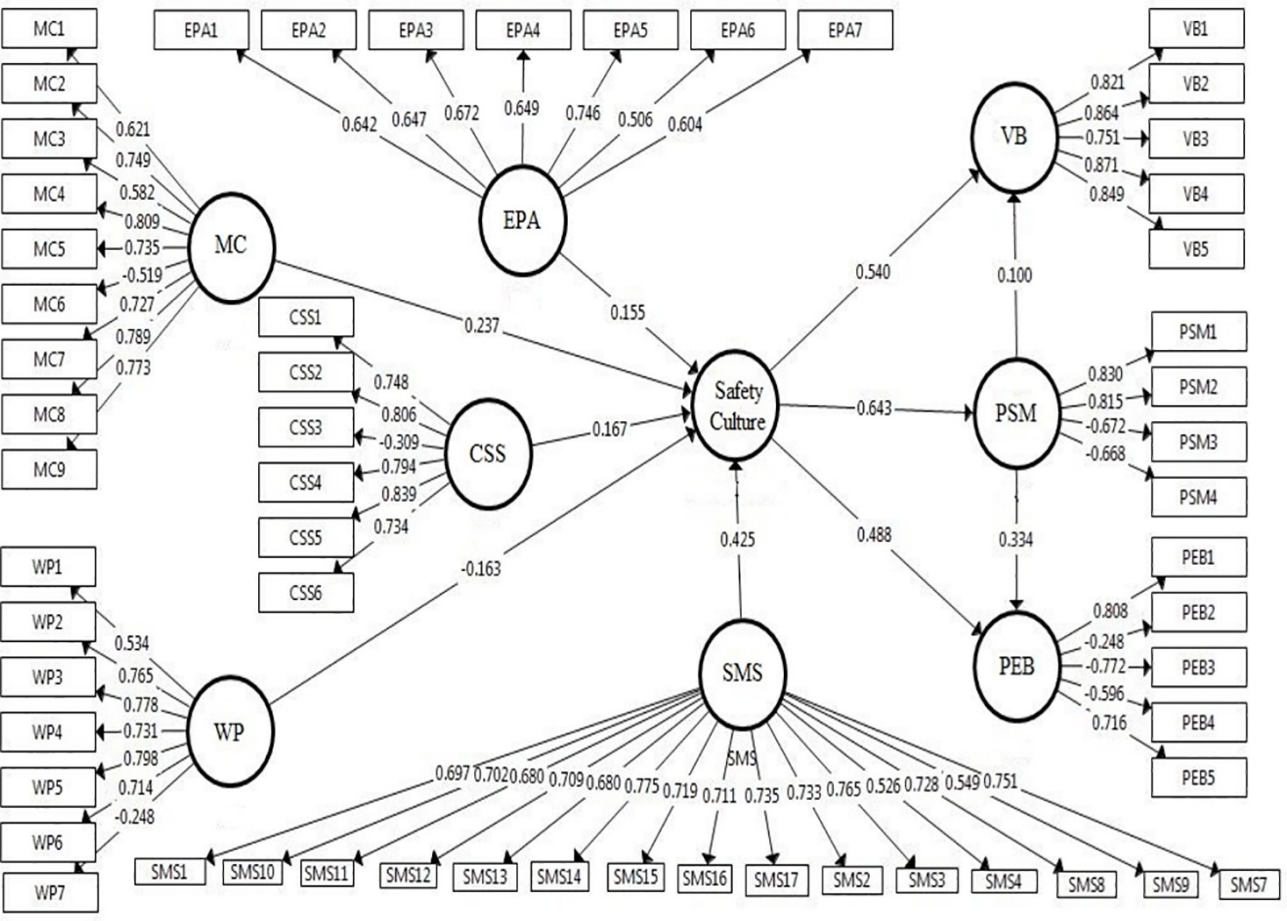
An initial structural model with standardized path coefficients.

The initial model consisted of some individual items having less than 0.50 loadings, which we removed and then reran the model (Fig 2). In total, we deleted one item from MC (MC6); two items from EPA (EPA 6 and EPA7); one item from CSS (CSS 3); two items from WP (WP 1, WP 7); one item from SMS (SMS 9); two items from PSM (PSM3 and PSM4); and three items from PEB (PEB2, PEB3, and PEB4). The results for the reliability and convergent validity of the revised model are given below (Table 4).

**Table 4:** Reliability and convergent validity: Comparison of the initial and final structural models.

	Number of items		Cronbach's Alpha		Average Variance Extracted (AVE)		Composite Reliability	
Constructs	Initial Model	Final Model	Initial Model	Final Model	Initial Model	Final Model	Initial Model	Final Model
**MC**	9	8	0.791	0.894	0.500	0.624	0.860	0.907
**EPA**	7	5	0.761	0.774	0.412	0.646	0.829	0.844
**CSS**	6	5	0.722	0.884	0.530	0.657	0.822	0.898
**WP**	7	5	0.729	0.841	0.460	0.611	0.814	0.884
**SMS**	15	14	0.925	0.954	0.491	0.623	0.915	0.936
**VB**	5	5	0.888	0.897	0.693	0.694	0.908	0.925
**PSM**	4	2	0.180	0.853	0.563	0.872	0.050	0.927
**PEB**	5	2	0.070	0.742	0.436	0.766	0.003	0.881

### SEM and bootstrapping test

The SEM approach was used to determine the degree to which the hypothesized model in this study was maintained and supported by the empirical data. SEM as a statistical method determines the relationships and directional influence, either direct or indirect, between the model’s latent variables, each of which has a set of observed variables in the conceptualized study model [54]. SEM has been commonly and successfully employed in most survey research in the behavioral and social sciences because of its ability to improve and validate the latent constructs or unobserved variables in measurement models [55]. The SEM methodology mainly consists of two parts: the measurement model and the structural model [55]. The structural model associates latent variables to measure the relationships between them, such as the direct and indirect effects, as well as the explained and unexplained variances accounted for in each latent variable [56]. In this study, SEM was used to the evaluate personnel safety culture in the petrochemical industry. Latent factors included endogenous variables of the petrochemical safety culture, personnel errors and behavior and personnel attitudes toward violation behaviors within the petrochemical environment. The analysis further employed petrochemical personnel safety motivation as a mediating variable.

The bootstrapping test is a resampling approach by which numbers of subsamples (5000 subsamples have been suggested mostly) are generated. The steps for its procedures include random sampling and replacing sets of samples from the actual data to obtain the subsamples; then, each subsample is applied to predict the model and achieve the partial least squares approach for the SEM (PLS-SEM) results. Later, these predictions are considered to acquire the distributions and are used to facilitate the significance tests [57].

### Model fit test

The PLS-SEM has no global goodness of fit index; thus far, the critical threshold values are not fully understood. Hence, the bootstrapping and blindfolding approaches are employed to address these problems [58]. In addition to these analyses, the reliability and validity tests for the measurement models are performed as an initial step [58]. The goodness of fit index is not usually reported; however, some researchers suggest the Standardized Root Mean Square Residual (SRMR) and Normed Fit Index (NFI) as performance metrics to assess model fit, which ensure the absence of model misspecification. Values of SRMR less than 0.10 or 0.08, and the closer that the NFI is to 1, the better the fit. NFI values greater than 0.9 usually represent acceptable fit [58]. In this study, the SRMR value is 0.053, which is less than the value of 0.08 that is considered acceptable. Moreover, the NFI value is approximately 0.92, which is considered a good fit for our model.

## Results

SEM was used to extract the structured model and to test the relationships among the study variables. Path analysis was employed by using each latent indicator to test the connections between each latent variable as well as the postulated hypotheses of the study.

A bootstrapping test was performed to assess the significance of the path coefficients using PLS-SEM depending on *t*-statistics and exerting the *t*-test values.

**Fig 3:**
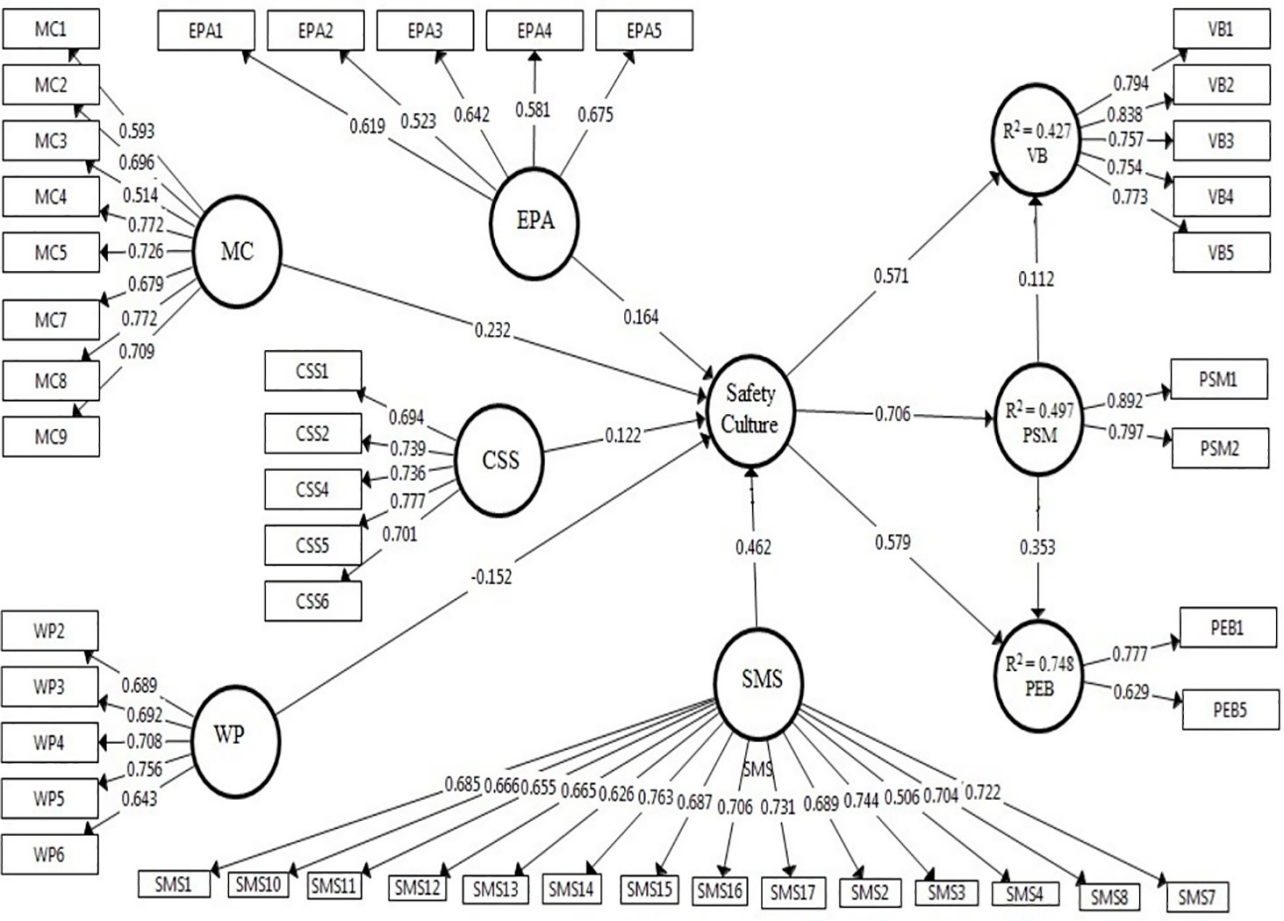
A final structural model with standardized path coefficients.

The estimated path coefficients and *t-*values between the latent variables are represented in Table 4. All hypotheses were supported by the survey results. The above analyses provide the following results (Fig 3):

- The perceived safety culture had a significant positive effect on personnel safety motivation in the petrochemical industry in Japan (standardized weight = 0.706; *p*-value < 0.05), which supports H1.
- A positive effect of the perceived safety culture on personnel error behaviors was identified in the petrochemical industry (β = 0.579; *p*-value < 0.05), which supports H2.
- The perceived safety culture positively influenced personnel attitudes toward violations in the petrochemical industry (β = 0.571; *p*-value < 0.05), which supports H3.
- Mediation existed only when the mediator variable had a significant effect on the dependent variable. In this study, the effect of personnel safety motivation on personnel error behavior in the petrochemical industry was statistically significant (β = 0.353; *p*-value < 0.05). Therefore, personnel safety motivation in these industries was found to mediate the relationship between the perceived safety culture and personnel error behaviors, which supported H4.
- Personnel safety motivation in the petrochemical industries mediated the relationship between the perceived safety culture and personnel attitudes toward behavior in relation to violations (β = 0.112; *p*-value < 0.05), which supports H5.

From the results of the hypothesis testing and bootstrapping, we can conclude that perceived safety culture plays a crucial role in personnel safety management. The change in perceived safety culture was found to affect the PSM, with R^2^ = 0.497. Similarly, the perceived safety culture and PSM play a critical role in PEB, with R^2^ = 0.748. That is, PEB was affected based on the perceived safety culture and PSM with a contribution of 74.8%. The perceived safety culture and PSM had an important role in VB, with R^2^ = 0.427 (Table 5 and Fig 3).

**Table 5:** Results of hypothesis testing.

Relationship	Std beta, β	*t*-statistics	*p*-value	Test result: hypothesis	R^2^
Perceived Safety Culture -> PSM	0.706	24.983	0.000*	H1: Supported	0.497
Perceived Safety Culture -> PEB	0.579	11.308	0.000*	H2: Supported	0.748
PSM -> PEB	0.353	6.215	0.000*	H4: Supported	
Perceived Safety Culture -> VB	0.571	12.864	0.000*	H3: Supported	0.427
PSM -> VB	0.112	2.176	0.000*	H5: Supported	

*Note: p-value was considered significant at the 0.05 level

## Discussion

The results of the present study offer several implications for assessing the perceived safety culture in Japan’s petrochemical industry. First, the perceived safety culture significantly affects personnel safety motivation. This finding demonstrates the need for assessing and enhancing the perceived safety culture in the petrochemical industry. This study confirmed the perceived safety culture’s predominant role as a predicting factor for enhancing personnel safety motivation. Second, the perceived safety culture was shown to significantly influence the formation of personnel safety behaviors. In Japan, personnel awareness in the petrochemical industry regarding safety culture is sufficient to influence error behaviors. In the examined petrochemical plants, the perceived safety culture had a direct and significant effect on personnel error and violation behaviors through personnel safety motivation.

These results revealed the need for management to reduce unsafe personnel conduct by improving safety procedures in daily routines. The study findings also highlight the need to examine safety management systems and ascertain organizational characteristics that directly or indirectly affect unsafe performance at work. Third, both personnel safety motivation and the perceived safety culture significantly affected personnel error behavior. Moreover, the perceived safety culture’s influence on personnel error demonstrated the mediating role of safety motivation. Fogarty and Shaw [15] examined the impact of management attitudes toward safety on maintenance personnel attitudes toward violations. The authors indicated that management awareness and support toward safety had a significant direct influence on the formation of personnel attitudes toward violations [15]. This result supports the findings of the present study, in which petrochemical personnel awareness regarding the safety culture in Japan was found to directly affect their own attitude toward violations.

The crucial, mediational role of safety motivation in the presented model is noteworthy. An appropriate perspective to discuss this issue is the classic theory of motivation, specifically, the goal setting theory [57,58,59]. According to this theory, a natural human tendency to determine and achieve goals is useful (effective) only when a given goal is understood and accepted. That is, the employee is motivated when he/she acts in a way that leads to the goal that he/she has accepted and deemed achievable. The goal-setting process itself is described classically as a four-phase process: 1) determining the pattern to achieve, 2) assessing whether this pattern can be achieved, 3) assessing pattern compliance with personal goals and 4) accepting the pattern, and thus setting a goal that leads to the actions/behaviors that lead to the goal [60]. It has long been known (e.g., [61]) that specific (clearly defined) goals with a high level of challenge are good motivators. It is also important that the challenge does not become stressful.

In contrast, the challenge should not be too easily attained. In both cases (challenge level too high or too low), the motivational function drops significantly. In practice, this means that safety motivation must balance between goals with a sufficiently high load of challenges that do not exceed the risk limit and goals bordering on monotony and boredom. Crossing these two boundaries, in the process of managing safety, usually leads to errors and/or accidents. In the safety management process, the explicit definition of goals is usually a smaller problem than defining and determining the suitable intensity of the level of challenge that accompanies these goals. The harmonious determination of both factors inside a perceived safety culture seems to be a key factor for safety motivation and, consequently, for safety at work. An additional factor that strengthens safety motivation is employee participation in goal setting activities [62]. In this case, the employees have an impact on the clarity of the specified goals and on the magnitude of the challenge load contained for these purposes.

Because the study used self-reported data collection through survey distribution, it is important to mention that the research participants might be influenced to report the general accepted safety procedure or conducts rather than stating their actual beliefs regarding each questions in the survey. In addition, this study did not evaluate relationships with objective indicators such as the rate of incidents and the number of reports by some type of error, and because the study design was cross-sectional, the causal relationships between variables are uncertain. Within this study, self-selection bias of participants may also have been a limitation as only those completed all survey statements were included in the final data set.

## Conclusion

This study investigated the relationships between a perceived safety culture and employee attitudes toward violations, error behavior and safety motivation in selected petrochemical plants of Japan. The proposed conceptual model demonstrated statistical significance among these relationships. The current research indicates that the perceived safety culture plays a role in petrochemical personnel safety motivation and safety performance. The findings of this study highlight the importance of perceived safety culture as a significant component of the organizational culture that influences employee behaviors and attitudes. These results may be used for future safety knowledge management that maximizes the use of employee safety knowledge in improving overall safety performance.

The outcomes of this study can provide significant contributions in helping managers in the petrochemical industry and governmental safety officials to improve worker safety motivation. Additionally, the results of this study can serve as a guide for adopting appropriate procedures to minimize worker error behavior and to augment attitudes toward violations in the petrochemical environment. In the future, additional research can investigate the main dimensions of perceived safety culture and determine which has the greatest impact on personnel safety performance. Future research is needed not only to refine and strengthen the findings and conclusions reached in this study but also to expand the scope of the factors considered here. There is an additional need to explore the impact of differences between the subcultures that are formed under general safety cultures within similar high-risk industries, such as construction, aviation, manufacturing and mining.
